# Efficacy of acupuncture as an adjunctive therapy for perimenopausal insomnia: A systematic review and meta-analysis of randomized controlled trials

**DOI:** 10.1016/j.clinsp.2025.100814

**Published:** 2025-10-28

**Authors:** Meiling Dong, Yunxia Sun, Xiaoman Wang, Xue Yu, Hanbin Li

**Affiliations:** a Acupuncture and Massage Department, Cangzhou Central Hospital, Cangzhou Hebei, China; b Traditional Chinese Medicine Clinic, Cangzhou Central Hospital, Cangzhou Hebei, China

**Keywords:** Adjunctive therapy, Acupuncture, Acupuncture therapy, Perimenopausal insomnia, Meta-analysis

## Abstract

**Objective:**

This investigation evaluates the efficacy of acupuncture used alongside conventional interventions for Perimenopausal Insomnia (PMI). The research aims to provide clinically relevant evidence, support individualized treatment planning, and improve sleep quality and overall well-being in perimenopausal women.

**Methods:**

A systematic search was conducted up to December 2024, encompassing six major databases, including international (PubMed, Web of Science, Cochrane Library, Embase) and Chinese-language sources (CNKI, Wanfang). The search focused exclusively on Randomized Controlled Trials (RCTs) investigating acupuncture as an adjunctive treatment for PMI. To evaluate result stability and identify potential heterogeneity sources, the authors performed sensitivity and subgroup analyses utilizing Review Manager (v5.4) and Stata (v15.0).

**Results:**

The study included 32 RCTs involving 2,673 participants. Compared with control interventions, the use of adjunctive acupuncture was associated with significantly improved therapeutic effects (RR = 1.25, 95 % CI [1.20, 1.30]). The observation group receiving adjunctive acupuncture therapy demonstrated significant advantages over the non-acupuncture group in improving: Pittsburgh Sleep Quality Index (PSQI) (SMD = -1.00, 95 % CI [-1.21, -0.79]), Menopause-Specific Quality of Life (MENQOL) (SMD = -0.56, 95 % CI [-0.88, -0.23]), Traditional Chinese Medicine Syndrome Score Scale (TCMSSS) (SMD = -1.40, 95 % CI [-2.55, -0.24]), Adverse Drug Reactions (ADR) (RR = 0.35, 95 % CI [0.17, 0.71]), Epworth Sleepiness Scale (ESS) (SMD = -0.57, 95 % CI [-0.83, -0.31]), and Insomnia Severity Index (ISI) (SMD = -0.94, 95 % CI [-1.43, -0.46]).

**Conclusion:**

Acupuncture as an adjunctive therapy enhances treatment outcomes for PMI, offering superior efficacy with minimal risks. Given the intrinsic limitations of the present study, additional investigations are necessary to substantiate these outcomes.

**Systematic Review Registration:**

PROSPERO database (ID: CRD420251020144).

## Highlights

• Acupuncture can enhance the clinical efficacy of PMI as an adjunctive therapy.
• The GV20 acupoint is the most widely used in PMI treatment.
• Acupuncture adjuvant treatment of PMI is safe and superior to medication.
• Individualized scheme design is flexible and effective.

## Introduction

Perimenopausal Insomnia (PMI) refers to sleep disturbances experienced by women during the transition to menopause. These disturbances are characterized by difficulties falling asleep, poor sleep quality, and frequent early awakenings.^1^ PMI often presents with additional symptoms, including hot flashes, night sweats, dizziness, and palpitations.^2^ Based on statistics from the World Health Organization (WHO), China currently has a population of around 160 million women in the perimenopausal stage ‒ the highest number globally. Projections indicate that this population could increase to 210 million by the year 2030.^3^ In China, the total prevalence of sleep disorders among women aged 40–65 is 39.1 %.^4^ Studies from other countries indicate varying rates: approximately 28 % among middle-aged women in South Korea,^5^ 46.2 % in Latin America,^6^ and 49.9 % in Iran.^7^ These high prevalence rates are concerning because chronic sleep deprivation significantly reduces the Quality of Life (QoL) for perimenopausal women by exacerbating negative emotions and impairing cognitive function. Additionally, it elevates the risks of developing obesity, diabetes, cardiovascular diseases, and mental health disorders.^8^^,^^9^

Currently, the primary Western medical treatments for PMI fall into two categories: pharmacological and non-pharmacological interventions. Among pharmacological interventions, Hormone Replacement Therapy (HRT)^10^ represents the mainstream treatment modality, which includes estrogen therapy, progestogen therapy, and combined estrogen-progestogen therapy.^11^ Non-hormonal pharmacological treatments include benzodiazepines and non-benzodiazepines. In clinical practice, the most commonly prescribed anxiolytics and sedative-hypnotics are benzodiazepines, such as diazepam, midazolam, estazolam, and lorazepam.^12^ While the therapeutic efficacy of Western medications for insomnia is well-established, they are also associated with numerous adverse effects.

Acupuncture is a vital component of Traditional Chinese Medicine (TCM). This approach involves stimulating specific acupoints through needling or moxibustion to regulate qi (vital energy) and blood, balance yin and yang, and thereby prevent or treat diseases. The WHO has recognized acupuncture for various conditions and includes insomnia among its indications. Both domestic and international guidelines, such as the Clinical Practice Guidelines for Acupuncture in China, recommend acupuncture as an adjunctive therapy for PMI.

Research has demonstrated that acupuncture increases the proportion of Slow-Wave Sleep (SWS), thereby improving sleep quality.^13^ Lu et al.^14^ found that acupuncture improves patients' sleep efficiency and the total effective rate of treatment. It also enhances sleep quality and improves daytime functioning.

A previous meta-analysis performed by Jiang et al.^15^ incorporated 14 studies and indicated that acupuncture is effective as an adjunctive treatment for PMI. The present study expanded the number of included studies to 32 and analyzed additional outcome measures. Building upon previous evidence, this analysis provides a more comprehensive and current assessment, confirming acupuncture's effectiveness in combination with standard treatment for PMI.

## Materials and methods

### Protocol registration and reporting standards

This meta-analysis was registered with the International Prospective Register of Systematic Reviews (PROSPERO, ID: CRD420251020144). Preferred Reporting Items for Systematic Reviews and Meta-Analyses (PRISMA) guidelines, its protocol framework, and the meta-analysis extension of PRISMA were strictly followed in reporting the present results.

### Search strategy

A structured search was conducted in six databases: PubMed, Web of Science, Cochrane Library, Embase, CNKI, and Wanfang, targeting Randomized Controlled Trials (RCTs) evaluating acupuncture as an adjunctive treatment for PMI. Search terms included “Acupuncture & Pharmacopuncture” and “Perimenopause & perimenopausal” and “Sleep Initiation and Maintenance Disorders & DIMS & Disorders of Initiating and Maintaining Sleep & Sleeplessness & Insomnia Disorder & Insomnia Disorders & Insomnia & Insomnias & Chronic Insomnia & Early Awakening & Nonorganic Insomnia & Primary Insomnia & Psychophysiological Insomnia & Rebound Insomnia & Secondary Insomnia & Sleep Initiation Dysfunction & Sleep Initiation Dysfunctions & Transient Insomnia” and “Random*”. No restrictions were placed on language or region. Additional relevant studies were identified through manual examination of reference lists from included articles. The complete search strategy is provided in Supplemental Table S1.

### Inclusion criteria and exclusion criteria

After screening articles through titles, abstracts, and full-text reviews, eligible RCTs were selected based on the following criteria:

Inclusion criteria:

a) Participants met the diagnostic criteria for perimenopausal syndrome in *Obstetrics and Gynecology* and for insomnia in the *Chinese Classification and Diagnostic Criteria for Mental Disorders* (CCMD-3) of the Psychiatric Branch of the Chinese Medical Association.^16^^,^^17^ 1) Eligible subjects were women aged 40–60 years who had been menopausal for 2-years or had experienced irregular menstruation for >3-months, accompanied by typical autonomic nervous symptoms such as irritability, chest tightness, dizziness, and tinnitus, as well as vasomotor manifestations including hot flashes, excessive sweating, and blood pressure fluctuations. 2) They also exhibited typical insomnia symptoms, including early awakening, light sleep with frequent awakenings, prolonged wakefulness, or severe insomnia throughout the night, accompanied by daytime functional impairment. Insomnia occurred at least three times per week and persisted for more than one month. 3) Participants had no diagnosed mental disorders or concurrent physical illnesses.
b) Interventions: The experimental group underwent acupuncture in addition to standard treatment, while the control group received standard treatment alone.
c) Outcomes: Studies reporting any of the following outcomes:
  • Primary outcomes: Clinical effectiveness rate or change in Pittsburgh Sleep Quality Index (PSQI).
  • Secondary outcomes: Changes in Menopause-Specific Quality of Life (MENQOL), Traditional Chinese Medicine Syndrome Score Scale (TCMSSS), Adverse Drug Reaction (ADR) rates, Epworth Sleepiness Scale (ESS), or Insomnia Severity Index (ISI).

Exclusion criteria:

a) Non-RCT study designs: Retrospective designs, reviews, and animal trials.
b) Cases of insomnia attributed to non-perimenopausal causes.
c) Studies where the intervention did not involve acupuncture.
d) Trials with flawed data reporting or incomplete outcome measurements.
e) Cases where original data could not be obtained from authors.
f) Duplicate publications.

### Data extraction

Two researchers (Dong ML and Wang XM) independently performed data extraction using the predefined eligibility criteria and search design. Extracted data encompassed authorship, publication year, study design, treatment course, participant demographics (sample size, age, intervention duration), intervention methods (techniques, durations, acupuncture points), and outcome variables. Continuous data were summarized as means and standard deviations, while categorical outcomes were recorded as event counts with corresponding totals. All outcome measures were derived from data collected at intervention completion. In cases of disagreement between reviewers, a third researcher (YX Sun) provided adjudication to resolve discrepancies.

### Quality evaluation

Risk of bias was appraised using the Cochrane Collaboration’s assessment tool, with two researchers (X Yu and HB Li) independently evaluating the quality of included RCTs. The assessment examined seven domains: random sequence generation, allocation concealment, blinding of participants and personnel, blinding of outcome assessment, completeness of outcome data, selective reporting, and other potential biases. Studies were classified into “low”, “high”, or “unclear” risk categories. A third researcher resolved any disagreements between the primary reviewers.

### Data analysis

Reference management was handled using EndNote 9.0, and structured data entry was performed in Excel. Analyses were executed using statistical platforms (Review Manager 5.4 and Stata 15.0). For dichotomous outcomes, Risk Ratios (RRs) were calculated; for continuous outcomes, results were expressed as Mean (MD) or Standardized Mean Differences (SMD).

Inter-study heterogeneity was tested using Cochran’s *Q* and the *I*^2^ statistic, with thresholds of p < 0.05 and *I*^2^ > 50 % used to guide model selection. Random-effects models were used where heterogeneity was substantial. A two-sided α level of 0.05 was adopted for statistical significance. Leave-one-out sensitivity analysis was performed to evaluate the influence of individual studies on the pooled effect size. Subgroup analyses were conducted, where data were sufficient, based on intervention methods, age groups, control conditions, and intervention duration to assess result robustness and identify potential sources of heterogeneity. Publication bias was examined via Egger's test.

## Results

### Literature screening

The database search yielded 636 records. After eliminating 231 duplicates, 405 records were screened by title and abstract, leading to the removal of 145 irrelevant studies. The remaining 260 studies underwent full-text assessment for eligibility, ultimately yielding 32 studies that met all inclusion criteria.^14^^,^^18^, ^19^, ^20^, ^21^, ^22^, ^23^, ^24^, ^25^, ^26^, ^27^, ^28^, ^29^, ^30^, ^31^, ^32^, ^33^, ^34^, ^35^, ^36^, ^37^, ^38^, ^39^, ^40^, ^41^, ^42^, ^43^, ^44^, ^45^, ^46^, ^47^, ^48^

**Fig. 1:**
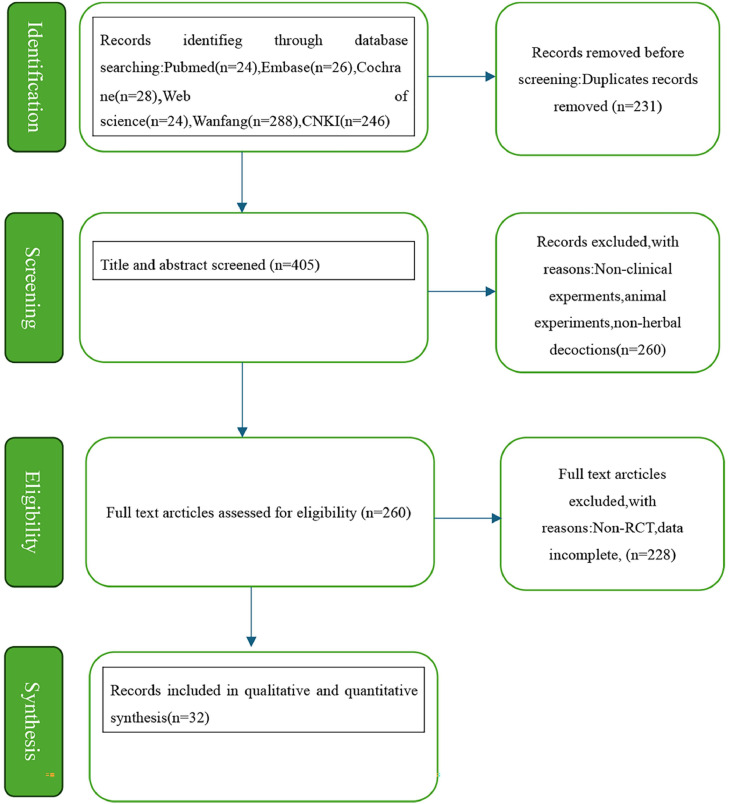
Flow plot.

The full selection process of eligible studies is illustrated in Fig. 1 using a PRISMA flow diagram.

### Study characteristics

The 32 included RCTs comprised 2,673 participants, with 1,365 receiving acupuncture in the intervention group and 1,308 assigned to the control group. All studies were conducted in China, with sample sizes exceeding 10 participants per group. Intervention durations ranged from 2 to 16 weeks. Control groups received conventional treatment or non-acupuncture interventions, while intervention groups received acupuncture as an adjunct to standard treatment. Detailed information on study populations, acupuncture protocols, and study designs is presented in Supplementary Table S2.

The studies utilized 62 different acupuncture points, with the ten most frequently used being: Baihui (GV20) on the Governor Vessel for treating headaches, dizziness, and insomnia; Shenmen (HT7) on the Heart Meridian for insomnia, anxiety, palpitations, and mental disorders; Sanyinjiao (SP6) at the intersection of the Liver, Spleen, and Kidney meridians for regulating menstruation, improving digestion, relieving pain, and treating insomnia; Sishencong (EX-HN1), four extra points surrounding Baihui for neurological and psychiatric disorders including insomnia, dementia, and headaches; Taixi (KI3) on the Kidney meridian for treating tinnitus, deafness, menstrual irregularities, heel pain, and diabetes; Yintang (EX-HN3) for insomnia, anxiety, and stress; Taichong (LR3) on the Liver Meridian for treating stroke, dizziness, menstrual disorders, dysmenorrhea, jaundice, hypochondriac pain, and urinary retention; Anmian (EX-HN22) primarily for insomnia, dizziness, headache, palpitations, and mental disorders; Shenting (GV24) for headache, dizziness, red and swollen eyes, lacrimation (excessive tearing), corneal opacity, night blindness, sinusitis, epistaxis (nosebleed), mania, epilepsy, and opisthotonos (back arching spasms); and Neiguan (PC6) for treating angina pectoris, myocarditis, arrhythmia, gastritis, and hysteria. Comprehensive details of all acupoints are available in Supplementary Table S3.

### Risk of bias assessment

**Fig. 2:**
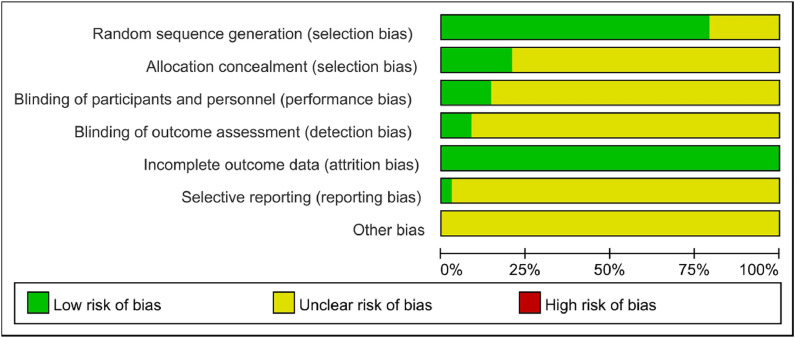
Risk of bias graph.

Study quality was assessed using the Cochrane risk-of-bias tool. Among the 32 studies, 25^45^^,^^48^, ^49^, ^50^, ^51^, ^52^, ^53^, ^54^, ^55^, ^56^, ^57^, ^58^, ^59^, ^60^, ^61^, ^62^, ^63^, ^64^, ^65^, ^66^, ^67^, ^68^, ^69^, ^70^, ^71^ used appropriate randomization methods, such as random number tables or dice rolling, and were rated as low risk for selection bias; the remainder did not clearly describe the randomization process and were rated as unclear. Seven studies^45^^,^^48^^,^^49^^,^^51^^,^^60^^,^^67^^,^^68^ used sealed envelope methods for allocation concealment (low risk), while others did not report this process (unclear risk). Only five studies^45^^,^^48^^,^^50^^,^^51^^,^^67^ reported blinding procedures and were considered low risk for performance and detection bias; the remainder lacked sufficient detail and were rated as unclear. All studies reported complete outcome data, indicating low risk for attrition bias. One study^48^ was prospectively registered and classified as low risk for reporting bias, whereas others, due to absence of registration, were rated as unclear. No additional sources of bias were identified, and overall risk was considered low, as illustrated in Fig. 2.

### Primary outcomes

#### Change in PSQI

**Fig. 3:**
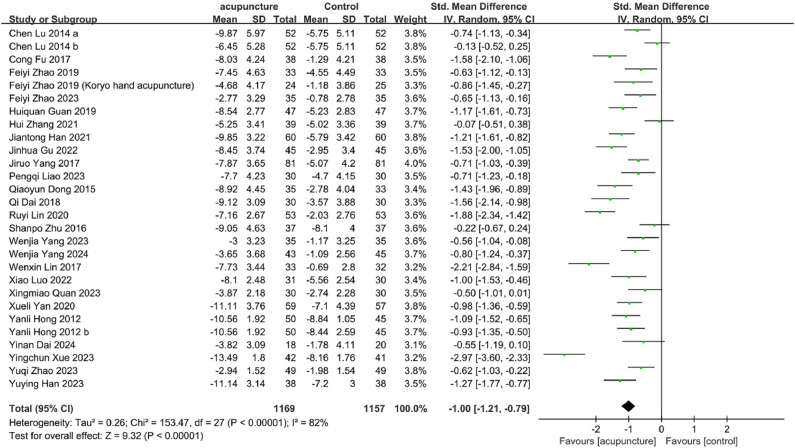
PSQI change value Forest plot.

Twenty-six studies^45^^,^^48^, ^49^, ^50^, ^51^^,^^53^, ^54^, ^55^, ^56^, ^57^, ^58^, ^59^, ^60^^,^^62^, ^63^, ^64^, ^65^, ^66^, ^67^^,^^69^, ^70^, ^71^, ^72^, ^73^, ^74^, ^75^ evaluated PSQI improvements. Results demonstrated that patients receiving acupuncture for PMI achieved significantly lower PSQI scores compared to control groups, indicating superior efficacy of acupuncture treatment (SMD = −1.00, 95 % CI [−1.21, −0.79], *I^2^* = 82 %, *p* < 0.00001) (Fig. 3).

Differences in effect were observed across intervention types in the subgroup analysis. Acupuncture alone (SMD = −0.87, 95 % CI [−1.15, −0.59], *I^2^* = 76 %, *p* < 0.00001), acupuncture plus non-pharmacological therapy (SMD = −1.03, 95 % CI [−1.57, −0.49], *I^2^* = 70 %, *p* = 0.0002), acupuncture plus TCM (SMD = −0.94, 95 % CI [−1.22, −0.65], *I^2^* = 70 %, *p* < 0.00001), and acupuncture plus Western medication (SMD = −1.41, 95 % CI [−2.30, −0.51], *I^2^* = 94 %, *p* = 0.002) all demonstrated notable improvements in PSQI scores compared to their respective control treatments. However, when compared specifically with zopiclone (a non-benzodiazepine hypnotic), acupuncture showed no significant advantage in improving PSQI scores (SMD = −0.55, 95 % CI [−1.19, 0.10], *p* = 0.10) (see details in Supplementary Table S4).

#### Clinical efficacy rate

**Fig. 4:**
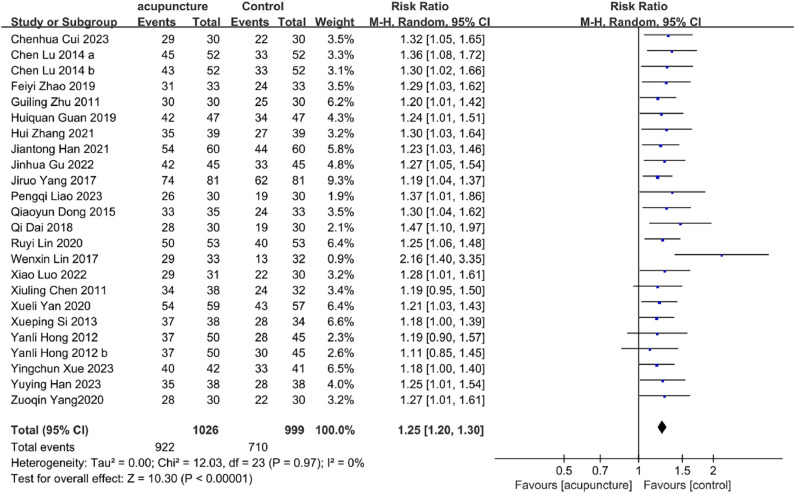
Clinical efficiency Forest plot.

Twenty-two studies^49^^,^^52^, ^53^, ^54^, ^55^, ^56^, ^57^, ^58^, ^59^, ^60^^,^^62^^,^^63^^,^^65^^,^^66^^,^^68^^,^^69^^,^^72^^,^^74^, ^75^, ^76^, ^77^, ^78^ reported clinical efficacy rates. Meta-analysis revealed that acupuncture as an adjunctive treatment for PMI achieved significantly higher efficacy rates relative to control interventions (RR = 1.25, 95 % CI [1.20, 1.30], *I*^2^ = 0 %, *p* < 0.00001) (Fig. 4).

Subgroup analysis consistently demonstrated benefits across all intervention types: acupuncture alone (RR = 1.27, 95 % CI [1.17, 1.38], *I^2^* = 20 %, *p* < 0.00001), acupuncture plus non-pharmacological therapy (RR = 1.26, 95 % CI [1.13, 1.40], *I^2^* = 0 %, *p* < 0.0001), acupuncture plus TCM (RR = 1.24, 95 % CI [1.16, 1.33], *I^2^* = 0 %, *p* < 0.00001), and acupuncture plus Western medication (RR = 1.23, 95 % CI [1.11, 1.36], *I^2^* = 0 %, *p* < 0.00001). The homogeneity of these findings (*I^2^* = 0 % across most comparisons) strengthens the reliability of these results. Complete subgroup analyses are available in Supplementary Table S3.

### Secondary outcomes

#### Change in menqol

**Fig. 5:**
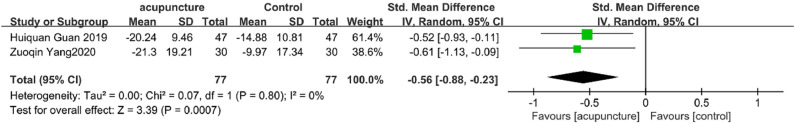
MENQOL score change value Forest plot.

Two studies^55^^,^^68^ reported changes in MENQOL scores. Acupuncture as an adjunctive treatment significantly reduced MENQOL scores compared to control interventions in patients with PMI (SMD = −0.56, 95 % CI [−0.88, −0.23], *I^2^* = 0 %, *p* < 0.001) (Fig. 5).

#### Change in tcmsss

**Fig. 6:**
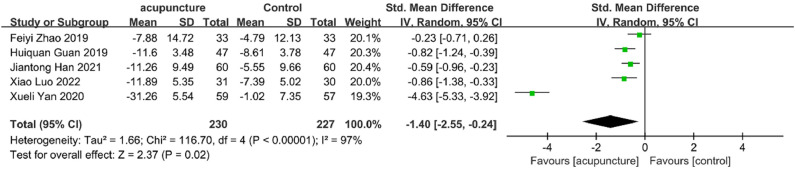
TCMSSS change value Forest plot.

Five studies^49^^,^^55^^,^^56^^,^^63^^,^^66^ examined TCMSSS outcomes. Meta-analysis revealed that adjunctive acupuncture significantly lowered TCMSSS scores compared to controls (SMD = −1.40, 95 % CI [−2.55, −0.24], *I^2^* = 97 %, *p* = 0.02) (Fig. 6).

#### ADR incidence

**Fig. 7:**
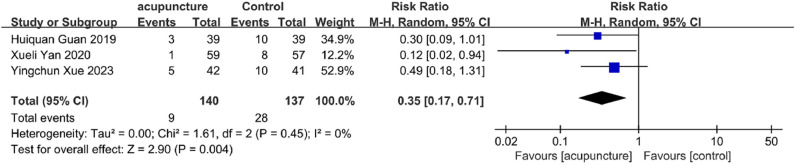
ADR change value Forest plot.

Three studies^55^^,^^65^^,^^66^ assessed ADR rates. Findings indicated that adjunctive acupuncture was associated with fewer adverse reactions compared to control treatments (RR = 0.35, 95 % CI [0.17, 0.71], *I^2^* = 0 %, *p* = 0.004) (Fig. 7), suggesting a superior safety profile.

#### Change in ESS

**Fig. 8:**
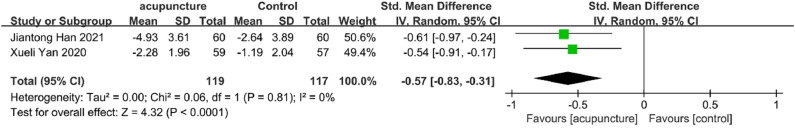
ESS change value Forest plot.

Two studies^56^^,^^66^ evaluated ESS outcomes. Patients receiving adjunctive acupuncture demonstrated greater reductions in daytime sleepiness as measured by ESS scores compared to controls (SMD = −0.57, 95 % CI [−0.83, −0.31], *I^2^* = 0 %, *p* < 0.0001) (Fig. 8).

#### Change in ISI

**Fig. 9:**
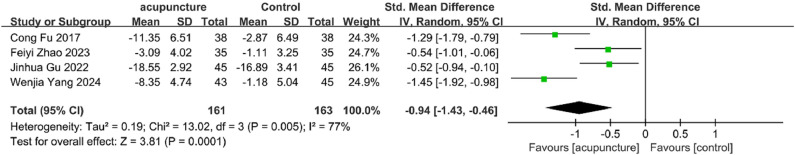
ISI score change value Forest plot.

Four studies^45^^,^^48^^,^^54^^,^^67^ analyzed ISI outcomes. Adjunctive acupuncture therapy yielded greater improvements in ISI scores than the control interventions (SMD = −0.94, 95 % CI [−1.43, −0.46], *I^2^* = 77 %, *p* = 0.0001) (Fig. 9).

### Publication bias and sensitivity analysis

Egger's test revealed evidence of publication bias for clinical efficacy rate (*p* = 0.0001), PSQI (*p* = 0.024), and TCMSSS (*p* = 0.0001). No significant publication bias was detected for ADR (*p* = 0.126) and ISI (*p* = 0.380).

**Fig. 10:**
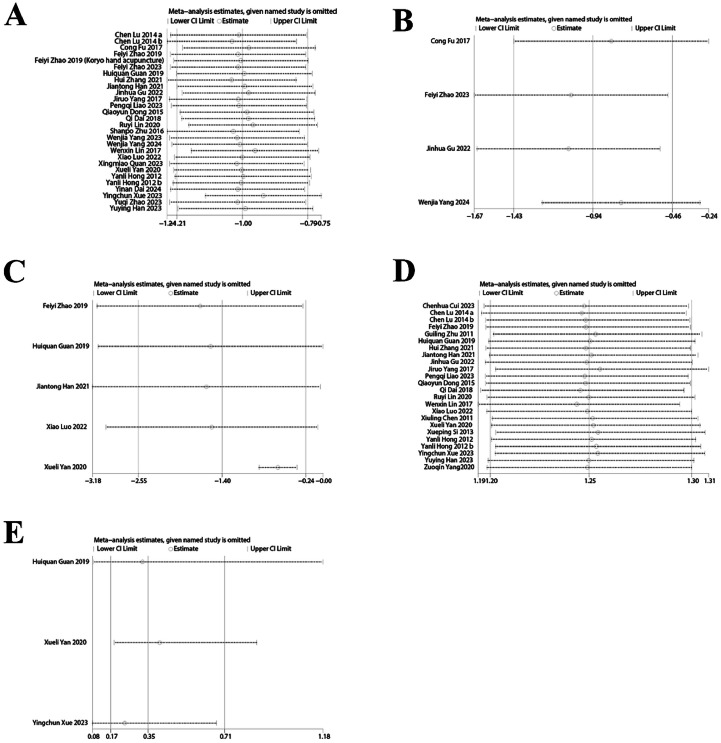
(A) PSQI change value sensitivity analysis; (B) ISI score change value sensitivity analysis; (C) TCMSSS change value sensitivity analysis; (D) Clinical efficiency sensitivity analysis; (E) ADR change value sensitivity analysis.

Sensitivity analyses for clinical efficacy rate, PSQI, ISI, and TCMSSS (Figs. 10A‒C) demonstrated that results remained consistent when individual studies were removed. However, the ADR results exhibited substantial instability when individual studies were sequentially omitted from the analysis, warranting caution in interpreting these findings (Fig. 10E).

## Discussion

Women aged 40 and above experience sleep disorders at approximately four times the rate of younger women.^79^ Among perimenopausal and menopausal women, the prevalence of insomnia increases significantly, ranging from 13.2 % to 65.1 %.^80^, ^81^, ^82^ PMI exerts significant adverse effects on women's physical and psychological well-being. Given these detrimental impacts, effective management of PMI is crucial for improving women's quality of life. Traditional Western medicine treatments have certain limitations, and acupuncture is a safe and useful non-pharmacological intervention option as an adjunctive therapy for PMI. As shown by Li et al.,^83^ the combination of acupuncture with TCM appears more effective than Western medicine in treating PMI, with higher efficacy rates (RR = 1.18; 95 % CI: 1.08, 1.29; *p* = 0.001) and greater improvement in PSQI scores (WMD = −2.77; 95 % CI: −4.15, −1.39; *p* < 0.0001). PMI falls under TCM syndromes such as “menopausal syndrome”, “sleeplessness”, “hysteria”, and “lily disease” (a condition characterized by emotional instability). Throughout history, medical practitioners have attributed insomnia in women at this stage to kidney deficiency and imbalance between yin and yang. Studies by Zhang et al.^84^ and Qin^85^ further demonstrated that acupuncture treatment groups showed superior results compared to control groups (oral estazolam tablets) across the Kupperman Menopausal Index (KMI), Hamilton Anxiety Scale (HAMA), and Hamilton Depression Scale (HAMD).

The meta-analysis confirms these previous findings, demonstrating that adjunctive acupuncture significantly improved clinical efficacy (Fig. 10D) and reduced scores on multiple assessment instruments, including PSQI, MENQOL, TCMSSS, ESS, and ISI (Fig. 10A‒10C) in perimenopausal women with insomnia. Additionally, acupuncture as an adjunctive treatment was associated with fewer ADRs compared to control interventions (Fig. 10E). The PSQI reduction (SMD = −1.00, equivalent to a 3.2-point decrease on the 0–21 scale) exceeds the established Minimal Clinically Important Difference (MCID) of 2.1 points for perimenopausal insomnia,^86^ indicating both statistical and clinical significance. Subgroup analyses suggest three populations derive maximal benefit: women receiving combined acupuncture-Western therapy, particularly those with refractory insomnia (PSQI SMD = −1.41 vs. monotherapy SMD = −0.87); patients with comorbid vasomotor symptoms, as acupuncture can mitigate hot flashes and other climacteric symptoms, thereby enhancing sleep improvement through synergistic pathways^87^; and patients with medication intolerance, particularly those with hepatic dysfunction.

Regarding acupuncture point selection, the analysis revealed that points used for treating PMI are predominantly located on the Governor Vessel (Du Mai), Heart Meridian, Pericardium Meridian, Spleen Meridian, Extraordinary Points (such as Sishencong), Stomach Meridian, Kidney Meridian, and Liver Meridian. The most frequently used acupoints included Baihui (GV20, used 23 times), Shenmen (HT7, 21 times), Sanyinjiao (SP6, 21 times), Sishencong (EX-HN1, 15 times), Taixi (KI3, 14 times), Yintang (EX-HN3, 13 times), Taichong (LR3, 13 times), Anmian (EX-HN22, 12 times), Shenting (GV24, 9 times), and Neiguan (PC6, 9 times). Two additional commonly used points were Guanyuan (CV4) and Zusanli (ST36), each used 9 times.

The treatment groups were stratified based on intervention modality. The analysis compared therapeutic outcomes ‒ including clinical efficacy rates and PSQI scores ‒ among four approaches: acupuncture monotherapy, acupuncture plus non-pharmacological therapy, acupuncture combined with TCM, and acupuncture with Western medication. Results demonstrated significant improvements across all intervention groups, confirming acupuncture's value as an effective adjunctive therapy for PMI. In the comparative analysis, the authors found no statistically significant difference between acupuncture and zopiclone regarding clinical efficacy and PSQI scores. However, this finding was based on a single study and requires further validation.

The therapeutic mechanisms of acupuncture in treating insomnia can be understood through multiple physiological pathways.^88^ These include neural signaling modulation, endocrine regulation, immune system effects, and gut microbiota interactions.

Neurotransmitter regulation represents a primary mechanism. Chen et al.^89^ demonstrated in animal models that acupuncture increases Hippocampal Serotonin (5-HT) levels in insomnia-model rats, promoting sleep without suppressing dopamine (DA) synthesis. Despite unchanged DA levels, acupuncture likely restores hippocampal DA quantity through complex regulatory pathways. The inhibitory neurotransmitter GABA (γ-aminobutyric acid) promotes deep SWS by suppressing neuronal activity in wakefulness-promoting brain regions. Clinical studies reveal that insomnia patients exhibit approximately 30 % lower GABA levels compared to healthy individuals.^90^ Acupuncture may alleviate insomnia by stimulating specific acupoints that influence hypothalamic neurons to upregulate GABA expression.^91^

Endorphin-mediated effects also contribute to acupuncture's efficacy. Cheng et al.^92^ found that electroacupuncture at the “Anmian” (EX-HN22) acupoint elevates β-endorphin concentrations in the brainstem and hippocampus, prolonging Non-Rapid Eye Movement (NREM) sleep in rats. This effect appears to involve cholinergic activation and μ-opioid receptor mediation.

Immune system modulation provides another mechanistic pathway. Cheng et al.^93^ determined that Tumor Necrosis Factor-Alpha (TNF-α) promotes 5-HT synthesis in brain tissue, increases 5-HT and its metabolite 5-Hydroxyindoleacetic Acid (5-HIAA), and regulates the sleep-wake cycle. Acupuncture elevates plasma TNF-α in insomnia-model rats, potentially improving sleep quality through this mechanism.

The gut-brain axis represents an emerging area of interest. Gut microbiota influences cognition and mood by regulating neurotransmitters, immune factors, and hormones.^94^ Probiotics in the digestive system modulate 5-HT secretion via the gut-brain axis, impacting sleep quality in insomnia patients.^95^ Han et al.^96^ demonstrated that acupuncture at Zusanli (ST36) and Tianshu (ST25) in rats rebalances gut microbiota, reducing Clostridium bifermentans while increasing Lactobacillus and Lachnospiraceae abundance.

Acupuncture has established both theoretical and empirical foundations for treating PMI, supported by neurophysiological research and evidence-based clinical studies. Its favorable safety profile, demonstrated efficacy, and capacity for personalized treatment contribute to its unique role in insomnia management. With ongoing mechanistic research and optimization of clinical protocols, acupuncture may become an increasingly important adjunctive therapy for PMI.

This meta-analysis encompassed 32 RCTs investigating acupuncture as an adjunctive treatment for PMI. As one of the most comprehensive analyses in this domain, the present results confirm acupuncture's efficacy and safety as an adjunctive intervention. The multidimensional assessment using validated instruments provides robust evidence to support clinical application and guide future research. However, several limitations should be acknowledged. All included studies were conducted in China with exclusively Chinese participants, leaving efficacy in non-Asian populations unverified. Considerable variability existed in intervention parameters, including needling depth (5–20 mm), retention time (20–40 min), and electroacupuncture frequency (2–100 Hz), which may have influenced outcomes. Outcome assessment timing varied from 2-weeks (capturing acute effects) to 16-weeks (evaluating sustained responses), limiting the establishment of optimal treatment duration. Furthermore, the TCMSSS scale lacks cross-cultural validation, potentially inflating effect estimates in settings with strong TCM orientation.

## Conclusion

Acupuncture as an adjunctive therapy for PMI demonstrates significant improvements in multiple outcome measures, including PSQI, MENQOL, TCMSSS, ESS, and ISI scores. Nevertheless, the geographical concentration of included studies in China, combined with their relatively small sample sizes and considerable heterogeneity, necessitates future international multicenter RCTs to further validate these findings. Such studies would establish more definitive evidence regarding the therapeutic efficacy and safety profile of acupuncture for PMI management.

## Funding

This work was supported by the Cangzhou Science and Technology Bureau project (grant No. 23244102077).

## Authors’ contributions

All authors contributed to the study conception and design. Writing-original draft preparation: Meiling Dong; Writing-review and editing: Yunxia Sun; Conceptualization: Xiaoman Wang; Methodology: Xue Yu; Formal analysis and investigation: Meiling Dong; Funding acquisition: Meiling Dong; Resources: Hanbin Li; Supervision: Yunxia Sun, and all authors commented on previous versions of the manuscript. All authors read and approved the final manuscript.

## Ethics approval and consent to participate

Not applicable.

## Consent for publication

Not applicable.

## Data availability statements

The original contributions presented in the study are included in the article/Supplementary Material.

## Declaration of competing interest

The authors declare no conflicts of interest.

## Appendix Supplementary materials

Image, application 1
