# Supplementary material for: Efficacy of acupuncture as an adjunctive therapy for perimenopausal insomnia: A systematic review and meta-analysis of randomized controlled trials
Source: Clinics (Sao Paulo). 2025 Oct 28;80:100814. doi: 10.1016/j.clinsp.2025.100814 (PMC12597306; doi:10.1016/j.clinsp.2025.100814)
Supplement: Supplementary file 1 [file mmc1.docx]

**CLINICS-D-25-00749_Supplementary Material**

**Supplemental** **Table S1 Search strategy.** Efﬁcacy of acupuncture for the adjunctive therapy of perimenopausal insomnia[: a systematic review](https://www.frontiersin.org/articles/10.3389/fphar.2024.1327030/full) [and meta-analysis of randomized](https://www.frontiersin.org/articles/10.3389/fphar.2024.1327030/full) [controlled trials](https://www.frontiersin.org/articles/10.3389/fphar.2024.1327030/full).

[Efﬁcacy of acupuncture for the adjunctive therapy of perimenopausal insomnia: a systematic review](https://www.frontiersin.org/articles/10.3389/fphar.2024.1327030/full) [and meta-analysis of randomized](https://www.frontiersin.org/articles/10.3389/fphar.2024.1327030/full) [controlled trials](https://www.frontiersin.org/articles/10.3389/fphar.2024.1327030/full)

Pharmacopuncture

DIMS

Disorders of Initiating and Maintaining Sleep

Sleeplessness

Insomnia Disorder

Insomnia Disorders

Insomnia

Insomnias

Chronic Insomnia

Early Awakening

Nonorganic Insomnia

Primary Insomnia

Psychophysiological Insomnia

Rebound Insomnia

Secondary Insomnia

Sleep Initiation Dysfunction

Sleep Initiation Dysfunctions

Transient Insomnia

| Pubmed-24 |
| --- |
| (((("Acupuncture"[Mesh]) OR (Pharmacopuncture)) AND (("Perimenopause"[Mesh]) OR (perimenopausal))) AND (("Sleep Initiation and Maintenance Disorders"[Mesh]) OR (((((((((((((((((DIMS) OR (Disorders of Initiating and Maintaining Sleep)) OR (Sleeplessness)) OR (Insomnia Disorder)) OR (Insomnia Disorders)) OR (Insomnia)) OR (Insomnias)) OR (Chronic Insomnia)) OR (Early Awakening)) OR (Nonorganic Insomnia)) OR (Primary Insomnia)) OR (Psychophysiological Insomnia)) OR (Rebound Insomnia)) OR (Secondary Insomnia)) OR (Sleep Initiation Dysfunction)) OR (Sleep Initiation Dysfunctions)) OR (Transient Insomnia)))) AND (Random*) |
| Embase-26 |
| 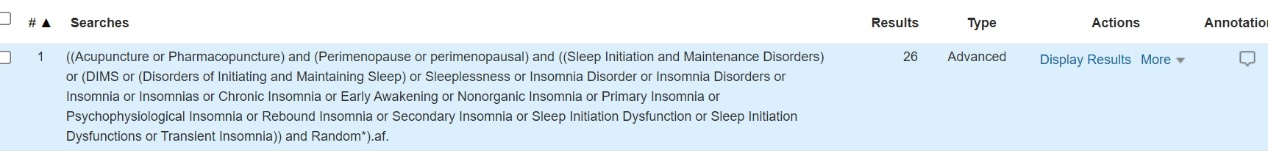 |
| Cochrane-28 |
| 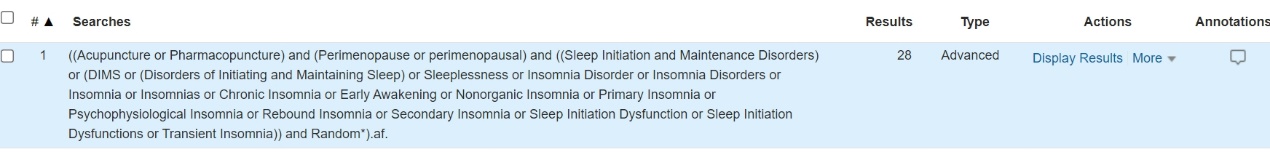 |
| WOS-24 |
| ((((Acupuncture) OR (Pharmacopuncture)) AND ((Perimenopause) OR (perimenopausal))) AND ((Sleep Initiation and Maintenance Disorders) OR (((((((((((((((((DIMS) OR (Disorders of Initiating and Maintaining Sleep)) OR (Sleeplessness)) OR (Insomnia Disorder)) OR (Insomnia Disorders)) OR (Insomnia)) OR (Insomnias)) OR (Chronic Insomnia)) OR (Early Awakening)) OR (Nonorganic Insomnia)) OR (Primary Insomnia)) OR (Psychophysiological Insomnia)) OR (Rebound Insomnia)) OR (Secondary Insomnia)) OR (Sleep Initiation Dysfunction)) OR (Sleep Initiation Dysfunctions)) OR (Transient Insomnia)))) AND (Random*) (Topic) |
| Wanfang-288 |
| 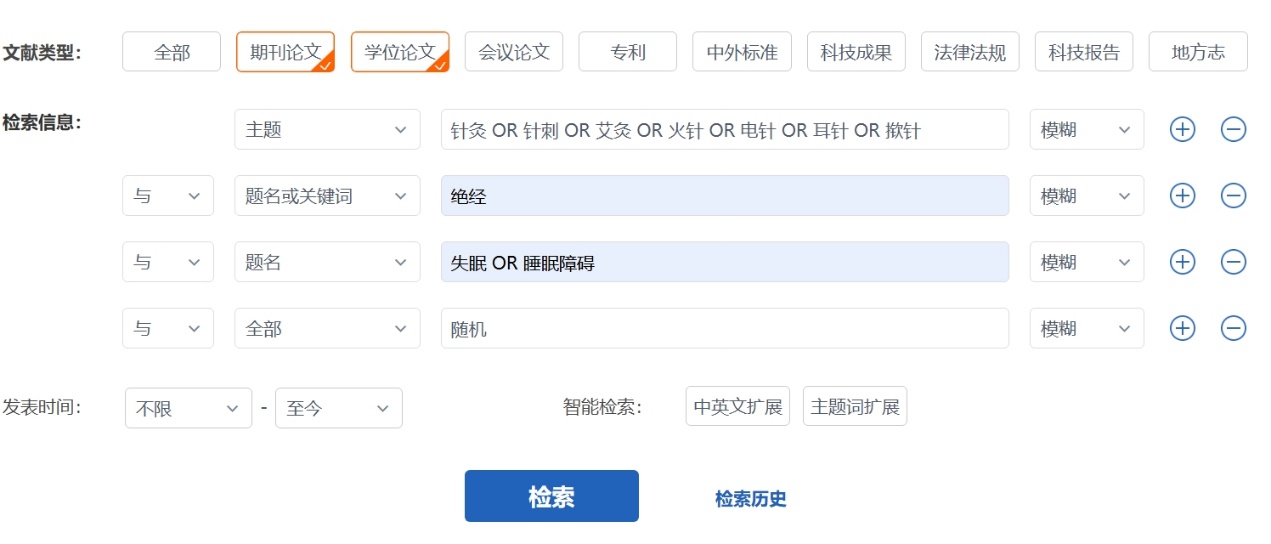 |
| CNKI-246 |
| 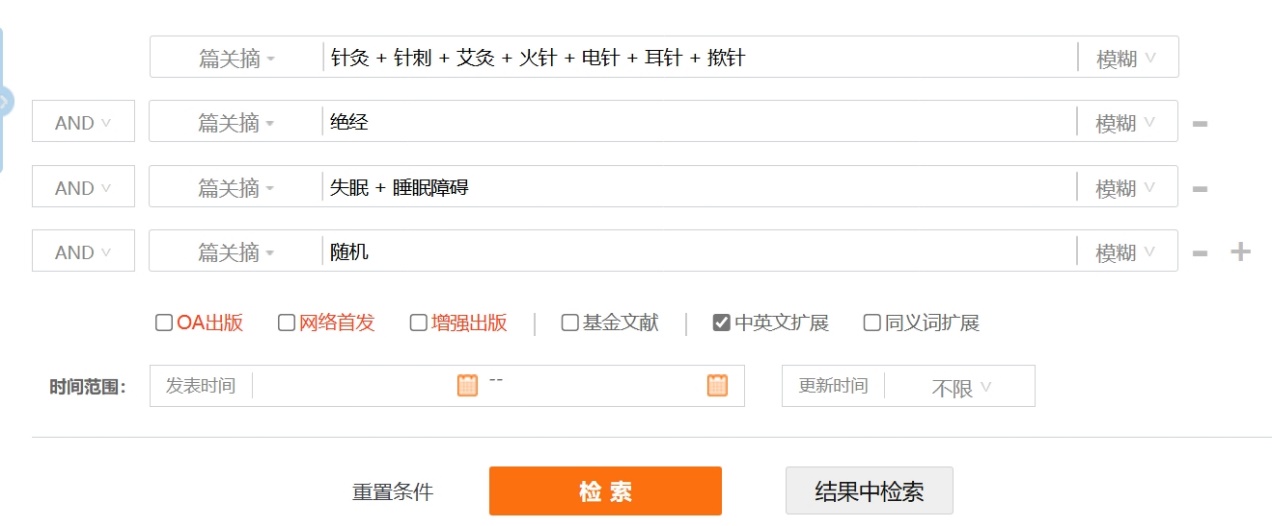 |

((((Acupuncture) OR (Pharmacopuncture)) AND ((Perimenopause) OR (perimenopausal))) AND ((Sleep Initiation and Maintenance Disorders) OR (((((((((((((((((DIMS) OR (Disorders of Initiating and Maintaining Sleep)) OR (Sleeplessness)) OR (Insomnia Disorder)) OR (Insomnia Disorders)) OR (Insomnia)) OR (Insomnias)) OR (Chronic Insomnia)) OR (Early Awakening)) OR (Nonorganic Insomnia)) OR (Primary Insomnia)) OR (Psychophysiological Insomnia)) OR (Rebound Insomnia)) OR (Secondary Insomnia)) OR (Sleep Initiation Dysfunction)) OR (Sleep Initiation Dysfunctions)) OR (Transient Insomnia)))) AND (Random*)

**Supplemental** **Table S2** Population details, acupuncture protocol, study design.

| **Author and year of publication** | **Study period** | **Region** | **Study design** | **Population details** | **Intervention Treatment (acupuncture protocol and other Treatment)** | **Control Treatment** | **Patients** | | **Intervention group time** |
| --- | --- | --- | --- | --- | --- | --- | --- | --- | --- |
|  |  |  |  |  |  |  | **Intervention group** | **Control group** |  |
| Huiquan Guan 2019 | 2016‒2018 | China | RCT | PMI | Acupuncture + traditional Chinese medicine: Yintang EX-HN3; anmian EX-HN22; Baihui GV20; Shenting GV24; sishencong EX-HN1; benshenBG13; Shenmen HT7; taixi KI3; Sanyinjiao SP6; Anshencumian: Dihuang, Huanglian, e Jiao, Shanyao, Huangqin, Duzhong, Gouqizi, Danggui, Suanzaoren, Yuanzhi, Fushen, Maidong, Baishao, Danshen, Baihe | Traditional Chinese medicine Anshencumian: Dihuang, Huanglian, e Jiao, Shanyao, Huangqin, Duzhong, Gouqizi, Danggui, Suanzaoren, Yuanzhi, Fushen, Maidong, Baishao, Danshen, Baihe | 47 | 47 | 4-weeks |
| Hui Zhang 2021 | 2019‒2020 | China | RCT | PMI | Acupuncture + traditional Chinese medicine: Baihui GV20, Shenmen HT7, Shenting GV24, Sishencong EX-1, Benshen BG13, Neiguan PC6, Sanyinjiao SP6; Baihe Dihuang Tang: Baihe, Shengdi, Suanzaoren, Zhimu Zhigancao | Estazolam | 39 | 39 | 4-weeks |
| Wenjia Yang 2023 | 2016‒2020 | China | RCT | PMI | Acupuncture: Baihui GV20, Shenshu BL23, Taixi KI3, Anmian EX-HN22 | Non-acupuncture | 35 | 35 | 4-weeks |
| Zuoqin Yang2020 | 2015‒2017 | China | RCT | PMI | Electroacupuncture + Auricular: Acupoints: Yintang EX-HN3, Baihui GV20, Neiguan PC6, Sanyinjiao SP6, Shenmen HT7, Taixi KI3, Hegu LI4, Taichong LR3 | Estazolam | 30 | 30 | 4-weeks |
| Xiuling Chen 2011 | 2008‒2010 | China | RCT | PMI | Acupuncture: Yingu KI10, FuliuKI7, Anmian EX-HN22, Shenmen HT7, Neiguan PC6, Taixi KI3, Taichong LR3, Baihui GV20, Yinlingquan SP9, Sanyinjiao SP6, Xuehai SP10, Xinshu BL15, Danshu BL19, Pishu BL20, Xingjian LR2, Taichong LR3, Fenglong ST40, Neiting ST44, Taixi KI3, Daling PC7, Geshu BL17, Danzhong CV17 | Estazolam | 38 | 32 | 5-weeks |
| Shanpo Zhu 2016 | 2013‒2015 | China | RCT | PMI | Acupuncture + Western medicine: Baihui GV20, Shenting GV24, Sishencong EX-HN1, Anmian EX-HN22, Shenmen HT7, Taichong LR3, Taixi KI3, Zhongwan CV12, Tianshu ST25, Yinlingquan SP9 + Estazolam | Estazolam | 37 | 37 | 4-weeks |
| Wenxin Lin 2017 | 2015‒2016 | China | RCT | PMI | Acupuncture: Qihai CV6, Guanyuan CV4, Zhongwan CV12, Baihui GV20, Shenting GV24, Yintang EX-HN3, Sanyinjiao SP6, Zusanli ST36, Neiguan PC6, Shenmen HT7 | Publicity and education | 33 | 32 | 4-weeks |
| Qi Dai 2018 |  | China | RCT | PMI | Acupuncture + moxibustion + Phlebotomy: Zusanli ST36, Hegu LI4, Taichong LR3, Baihui GV20, Yintang EX-HN3, Guanyuan CV4, Zhongwan CV12; Phlebotomy: Dazhui GV14, Xinshu BL15, Geshu BL17; Moxibustion: Dazhui GV14, Xinshu BL15, Shenshu BL23, Mingmen GV4, Guanyuan CV4 | Estazolam | 30 | 30 | 4-weeks |
| Yuying Han 2023 | 2021‒2022 | China | RCT | PMI | Acupuncture: Qiha iCV6, Guanyuan CV4, Zhongwan CV12, Xiawan CV10, Shuifen CV9, Tianshu ST25, Huaroumen ST24, Daheng SP15, Zigong EX-CA1, Daimai GB26, Yintang EX-HN3, Shenting GV24, Benshen BG13, Sishencong EX-HN1, Shenmen HT7, Sanyinjiao SP6, Zhaohai, Shenmai, Qimen, Taichong | Estazolam, Gu Wei Su | 38 | 38 | 8-weeks |
| Yingchun Xue 2023 |  | China | RCT | PMI | Acupuncture + Western medicine: Sishencong EX-HN1, Anmian EX-HN22, Baihui GV20, Shenmai BL62, Hegu LI4, Fenglong ST40, Qimen LR14, Xingjian LR2, Taichong LR3, Ganshu BL18, Zhaohai KI6, Sanyinjiao SP6, Zusanli ST36; Estazolam | Estazolam | 42 | 41 | 4-weeks |
| Pengqi Liao 2023 | 2021‒2022 | China | RCT | PMI | Acupuncture: yintang SX-HN3, Yangbai GB14, Guanyuan CV4, Guilai ST29, Sanyinjiao SP6, Qihai CV6, Neiguan PC6, Shenmen HT7, Laogong PC8, Yongquan KI1, Quanzhong, Quannei | Alprazolam | 30 | 30 | 2-weeks |
| Chen Lu 2014 | 2010‒2013 | China | RCT | PMI | Acupuncture: group 1: Sishencong EX-HN1, Baihui GV20, Fengchi GB20, Shenmen HT7, Zhongwan CV12, Daheng SP15, Qimen LR14, Sanyinjiao SP6, Taichong LR3; Group 2: Baihui GV20, fengfu GV16, Shenting GV24, Yintang EX-HN3, Dazhui GV14 | Estazolam | 52 | 52 | 30 |
| Jiruo Yang 2017 | 2014‒2016 | China | RCT | PMI | Acupuncture: Neiguan PC6, Shenmen HT7, Zusanli ST36, Taixi KI3, Fenglong ST40, Zhongwan CV12: Forehead belt MS1, Middle of the rated band 1/3, Rear of the rated belt 1/3, The front of the frontal basement band 1/3, 1/3 of the middle of the skull base band | Estazolam | 81 | 81 | 3-month |
| Ruyi Lin 2020 | 2018‒2019 | China | RCT | PMI | Acupuncture + Western medicine: Qihai CV6, Xiawan CV10, Zhongwan CV12, gGuanyuan CV4; moxibustionyongquan KI1 + Gu Wei Su | Gu Wei Su | 53 | 53 | 20-days |
| Xueli Yan 2020 | 2018.1‒12 | China | RCT | PMI | Acupuncture + traditional Chinese medicine: Sishencong SX-HN1, Anmian EX-HN22, Shenmen HT7, Sanyinjiao SP6; Ganshu BL18, Feishu BL13, Fengchi GB20, Zusanli ST36; Xiangfu tang | Estazolam | 59 | 57 | 16-weeks |
| Yuqi Zhao 2023 | 2020‒2022 | China | RCT | PMI | Acupuncture + Auricular: Baihui GV20, Shenting GV24, Lieque LU7, Quchi LI11, Neiguan PC6, Hegu LI4, Fengchi GB20, Fengfu GV16, Qihai RN6, Guanyuan RN7, Weizhong BL40, Sanyinjiao SP6, Fenglong ST40; Auricular: Shenmen, sympathy, Subcortical, heart, liver, kidney | Estazolam | 49 | 49 | 4-weeks |
| Jiantong Han 2021 | 2016‒2018 | China | RCT | PMI | Acupuncture + traditional Chinese medicine: medicinal herb: Guyuan Ningshen Tang + Acupoints: Xinshu BL15, Pishu BL20, Shenshu BL23, Baihui GV20, Shenting GV24, Fengfu GV16, Shenmen HT7, Zusanli ST36, Sanyinjiao SP6, Zhaohai KI6, Taixi KI3, Taichong LR3 | Medicinal herb:gu yuan ning shen tang | 60 | 60 | 2-weeks |
| Xiao Luo 2022 | 2020‒2021 | China | RCT | PMI | Acupuncture + Gua Sha: Yintang EX-HN3, Baihui GV20, Shenmen HT7, Anmian EX-HN22, Shenmai BL62, dadun LR1, Taichong LR3, Yingu KI10 + DU Meridian, bladder meridian of foot-Taiyang | Gua Sha DU Meridian, DU bladder meridian of foot-taiyang, BL | 31 | 30 | 4-weeks |
| Xueping Si 2013 | 2012‒2013 | China | RCT | PMI | Acupuncture + Auricular: Taixi KI3, Sanyinjiao SP6, Baihui GV20, Sishencong EX-HN1, Shenmen HT7, Shenmai BL62, Zhaohai KI6 + Auricular; heart, kidney, liver, shenmen, incretion, sympathy | Estazolam | 38 | 34 | 4-weeks |
| Jinhua Gu 2022 | 2019‒2020 | China | RCT | PMI | Acupuncture + Western medicine + Music: Baihui GV20, Shangxing GV23, Yintang EX-HN3, Anmian EX-HN22, Shenmen HT7, heguLI4, Zusanli ST36, Sanyinjiao SP6 + Estazolam + tone of Jiao, tone of yu | Estazolam | 45 | 45 | 4-weeks |
| Guiling Zhu 2011 | 2009‒2010 | China | RCT | PMI | Acupuncture: Yintang EX-HN3, Baihui GV20, Sishencong EX-HN1, Shenmen HT7, Neiguan PC6, Anmian EX-HN22 | Gu Wei Su | 30 | 30 | 30-days |
| Wenxin Lin 2022 | 2019‒2020 | China | RCT | PMI | Acupuncture + traditional Chinese medicine: Baihui GV20, Yintang EX-HN3, Sishencong EX-HN1, Sanyinjiao SP6, Zusanli ST36, Qihai CV6, Guanyuan CV4, Tianshu ST25, Zhongwan CV12, Xiawan CV10, Shenmen HT7, Taichong LR3 + traditional Chinese medicine: Qingre Anshen Tang | Alprazolam | 50 | 47 | 2-mouth |
| Qiaoyun Dong 2015 | 2013‒2014 | China | RCT | PMI | Acupuncture + traditional Chinese medicine: Baihui GV20, Sanyinjiao SP6, Shenmen HT7, Sishencong EX-HN1, Taixi KI3, Taichong LR3 + Huang Liane Jiao Tang | Huang Lian e Jiao Tang | 35 | 33 | 20-days |
| Yanli Hong 2012 | 2010‒2011 | China | RCT | PMI | Electroacupuncture + traditional Chinese medicine group: Anmian EX-HN22, Sishencong EX-HN1, Shenmen HT7, Sanyinjiao SP6, Shenmai BL62, Zhaohai KI6; Taixi KI3, Daling PC7 + traditional Chinese medicine: Yishen Qinggan tang | Traditional Chinese medicine: Yi Shen Qing Xin Tang Electroacupuncture: Anmian EX-HN22, Sishencong EX-HN1, Shenmen HT7, Sanyinjiao SP6, Shenmai BL62, Zhaohai KI6; Taixi KI3, Daling PC7 | 50 | 45 | 16-weeks |
| Chenhua Cui 2023 | 2020‒2021 | China | RCT | PMI | Acupuncture + traditional Chinese medicin: Zi Shui Qing Gan Tang + Baihui GV20, Yintang EX-HN3, Sishencong EX-HN1, Tianshu ST25, Qihai CV6, Guanyuan CV4, Taichong LR3, Hegu LI4, Sanyinjiao SP6, Zusanli ST36 | traditional Chinese medicine: Zi Shui Qing Gan Yin | 30 | 30 | 4-weeks |
| Xingmiao Quan 2023 | 2019‒2022 | China | RCT | PMI | B group: Estazolam + Qinzhen: Shenshu BL23, Xinshu BL15, Sanyinjiao SP6, Taixi KI3, Fuliu KI7, Shenmen HT7 C group: Zishui Qing Gan Tang + Estazolam D group: Estazolam + Qinzhen + Zishui Qing Gan Tang | A group: Estazolam | 30 | 30 | 2-weeks |
| Yinan Dai 2024 | 2023.2‒2023.8 | China | RCT | PMI | Acupuncture: Baihui GV20, Zhaohai KI6, Shenmai BL62, Shenshu BL23, Aanyinjiao SP6, Anmian EX-HN22, Taixi KI3 | Western medicine: Zopicron | 18 | 20 | 4-weeks; 2-weeks |
| Feiyi Zhao 2023 | 2023.2‒2023.12 | China | RCT | PMI | Acupuncture: Yintang EX-HN3, Baihui GV20, Guanyuan CV4, Yinjiao CV7, Neiguan PC6, Taixi KI3, Taichong, LR3 Sanyinjiao SP6 Zigong, EX-CA1 | Non-acupuncture: Zhouliao LI12, Shouwuli LI13, Tiaokou ST38, Yangfu GB38, Xuanzhong GB39, Sanyangluo TE8, Sidu TE9 | 35 | 35 | 8-weeks |
| Cong Fu 2017 | 2015‒2016 | China | RCT | PMI | Acupuncture: Shenshu BL23, Ganshu BL18, Qimen LR14, Jingmen GB25 | Non-acupuncture: placebo acupuncture | 38 | 38 | 2-weeks |
| Wenjia Yang 2024 |  | China | RCT | PMI | Acupuncture: Shenshu BL23, Taixi KI3, Baihui GV20, Anmian EX-HN22 | Non-acupuncture | 43 | 45 | 4-weeks |
| Feiyi Zhao 2019 | 2015‒2016 | China | RCT | PMI | Acupuncture + traditional Chinese medicine: Luohuaanshen Tang + Electroacupuncture: Sishencong EX-HN1, Shenting GV24, Benshen GB13, Baihui GV20, Shenmai BL62, Zhaohai KI6 | Luohuaanshen tang | 33 | 33 | 4-weeks |
| Feiyi Zhao 2019 |  | China | RCT | PMI | Korean hand acupuncture: F6 (an acupoint known to control gynecological disorders), C7 (an acupoint known to be involved in insomnia), G15 and N1 (two acupoints known to regulating sympathetic functions and relieve anxiety, sweating, fatigue, hot ﬂashes, and blood circulation disorders), and A28 and B19 points (two acupoints known to stimulate the hypothalamus and regulate the release of hormones | Non-hand acupuncture | 24 | 25 | 8-weeks |

**Supplemental** **Table S3** Acupuncture point information sheet.

| **Acupoint pinyin** | **International code** | **Effect** | **Frequency** |
| --- | --- | --- | --- |
| baihui | GV20 | Stomach prolapse/post-stroke sequelae/lightheadedness/headcche/insomnia/anxiety/diarrhea | 23 |
| shenmen | HT7 | Insomnia/anxiety/depression, and stress/PTSD, panic attacks, and emotional instability/wrist pain, carpal tunnel syndrome, and tendonitis/high blood pressure | 21 |
| sanyinjiao | SP6 | Irregular periods, dysmenorrhea/digestive issues like bloating, diarrhea/insomnia/ anxiety | 21 |
| sishencong | EX-HN1 | Improves memory, concentration, and cognitive abilities/Helps relieve insomnia, anxiety, and mental fatigue/ Dementia, post-stroke recovery, epilepsy | 15 |
| taixi | KI3 | Night sweats/tinnitus/dizziness/lower back pain/enuresis/frequent urination/chronic nephritis/insomnia/sore throat/heel pain | 14 |
| yintang | EX-HN3 | Lightheadedness/headcche/insomnia/anxiety/rhinitis/eye strain | 13 |
| taichong | LR3 | Anxiety, depression, insomnia/Irregular menstruation, dysmenorrhea/Migraines, glaucoma, blurred vision, chronic sore throat/Hypertension, pediatric convulsions, hiccups | 13 |
| anmian | EX-HN22 | Improve insomnia/calms the mind/regulate nervous system/relieves tinnitus | 12 |
| shenting | GV24 | Relieve anxiety/insomnia/neurasthenia/headcche/rhinitis/eye strain | 9 |
| neiguan | PC6 | Palpitations, chest tightness/anxiety, insomnia/nausea, vomiting, motion sickness/headache, arm pain, carpal tunnel syndrome | 9 |
| guanyuan | CV4 | Erectile dysfunction, prostatitis/PCOS, endometriosis, menopausal symptoms/Chronic diarrhea, abdominal coldness/Fatigue, cold intolerance, post-illness recovery | 9 |
| zusanli | ST36 | IBS, gastritis, ulcers, poor appetite/Fatigue, anemia, post-surgery recovery/Arthritis, muscle atrophy, sports injuries | 9 |
| zhongwan | CV12 | Gastroparesis (delayed gastric emptying) / Irritable Bowel Syndrome (IBS) / Gastroesophageal Reflux Disease (GERD) / Chronic Fatigue (due to poor nutrient absorption) | 8 |
| shenshu | BL23 | Cold limbs, low back pain, fatigue, edema, frequent urination, impotence/night sweats, dizziness, tinnitus, dry throat, insomnia/chronic fatigue, adrenal exhaustion | 7 |
| qihai | CV6 | Chronic fatigue syndrome/Low blood pressure/anaemia/Bronchial asthma in remission/infertility/Decreased sexual function/Menopausal syndrome/enuresis | 7 |
| zhaohai | KI6 | Insomnia/Anxiety disorders/cystitis/Urinary tract infections/Menopausal syndrome/Menstrual bleeding is light/Chronic pharyngitis/Vocal cord nodules | 7 |
| shenmai | BL62 | Ankle sprains/sciatica/Rehabilitation from paralysis of the lower limbs/Adjuvant treatment for epilepsy/Narcolepsy/The eyes are red, swollen and painful/Eyebrow bone pain | 7 |
| hegu | LI4 | Headaches, toothaches, menstrual pain, and post-surgical pain/anxiety, and facial paralysis/TMJ disorders, toothache, sinusitis, and facial swelling/Induces Labor | 6 |
| benshen | GB13 | Insomnia/anxiety/headcche/lightheadedness/facial paralysis | 4 |
| xinshu | BL15 | Adjuvant treatment for coronary heart disease/Cardiac neurosis/hypertension/Nervous breakdown/depression/Menopausal syndrome/Night sweats/forgetful/Sore tongue | 4 |
| fenglong | ST40 | Gastritis/Esophageal reflux/Hyperlipidemia/Meniere's disease/Adjuvant treatment for epilepsy/Simple obesity/Hyperlipidemia | 4 |
| tianshu | ST25 | Irritable bowel syndrome/Chronic colitis/Adjuvant treatment for appendicitis/Pelvic inflammatory disease/endometritis/Simple obesity/Hyperlipidemia | 4 |
| qimen | LR14 | Chronic hepatitis/cholecystitis/Gastroesophageal reflux/depression/Anxiety disorders/Menopausal syndrome/Breast hyperplasia/Premenstrual tension syndrome/Irregular menstruation | 4 |
| xiawan | CV10 | Chronic gastritis/gastroptosis/Functional dyspepsia/corpulent/Diabetic gastroparesis/Pediatric food accumulation/Postoperative gastrointestinal function recovery | 3 |
| fengchi | GB20 | Allergic rhinitis/Chronic sinusitis/migraine/Meniere's disease/insomnia/Cervical spondylosis/scapulohumeral periarthritis/glaucoma/tinnitus | 3 |
| fengfu | GV16 | Sequelae of stroke/Vascular dementia/epilepsy/schizophrenia/Anxiety disorders/Loss of voice/The tongue is strong and silent/Nuchal rigidity/Scalp numbness | 3 |
| ganshu | BL18 | Chronic hepatitis/cholecystitis/Cholelithiasis/depression/insomnia/migraine/Breast hyperplasia/Premenstrual syndrome/infertility/glaucoma/conjunctivitis | 3 |
| yingu | KI10 | Prostatitis, urinary tract infections, enuresis/Dysmenorrhea, infertility, leukorrhea/Medial knee pain, stiffness, or weakness/Low-grade fever, night sweats, restlessness. | 2 |
| fuliu | KI7 | Chronic nephritis / Urinary tract infections / Frequent nocturia / diabetes / Hyperthyroidism / Functional uterine bleeding/prostatitis/osteoporosis / Tinnitus and deafness | 2 |
| yinlingquan | SP9 | Nephritis edema/cystitis/Prostatic hyperplasia/enteritis/dyspepsia/hepatitis/Pelvic inflammatory disease/Hydrosalpinx/Menopausal syndrome/Arthritis of the knee/Gouty arthritis | 2 |
| pishu | BL20 | Chronic gastritis / Irritable bowel syndrome / Peptic ulcers/Iron deficiency anemia / Thrombocytopenic purpura / Functional uterine bleeding / Postpartum weakness/diabetes/Adjunctive myasthenia gravis/Pediatric chancre accumulation | 2 |
| hangjian | LR2 | Hypertension/migraine/Facial spasms/Adjuvant treatment for mania/Menopausal syndrome/conjunctivitis/glaucoma/tinnitus/mastitis/dysmenorrhea | 2 |
| daling | PC7 | Insomnia/anxiety/palpitation/gastralgia/tenosynovitis/Wrist pain | 2 |
| geshu | BL17 | Intercostal neuralgia/dysmenorrhea/amenorrhoea/Postpartum abdominal pain/Blood in the stool/anaemia/belch | 2 |
| dazhui | GV14 | Cold/fever/Cervical spondylosis/ankylosing spondylitis/Stiff neck/insomnia/epilepsy | 2 |
| daheng | SP15 | Acute enteritis/Chronic enteritis/Postoperative intestinal paralysis/Pediatric chancre accumulation/corpulent | 2 |
| zigong | EX-CA1 | Amenorrhoea/dysmenorrhea/Menstrual irregularities/infertility/Uterine prolapse/Pelvic inflammatory disease/Uterine fibroids/Ovarian cysts/Urine retention | 2 |
| yongquan | KI1 | Insomnia/anxiety/hypertension/sunstroke/Febrile seizures/enuresis | 2 |
| xuehai | SP10 | Menstrual irregularity/Uterine fibroids/Ovarian cyst/Postpartum lochia/ Anemia/Hives / Urticaria/Eczema/Knee joint pain | 1 |
| danshu | BL19 | Cholecystitis/gallstones/and biliary dyskinesia/insomnia/migraines/intercostal neuralgia | 1 |
| neiting | ST44 | Toothache/gingivitis/bad breath/ stomachache/acid reflux/nausea/vomiting/abdominal distension/ restlessness/insomnia/foot pain | 1 |
| danzhong | CV17 | Stress/ irritability / insomnia/Angina/coronary heart disease/costal pain/Cough/Poor milk supply/mastitis | 1 |
| mingmen | GV4 | Low back pain/impotence / infertility/frequent urination/sciatica/ irregular periods/menstrual cramps/infertility/Osteoarthritis/spinal stiffness | 1 |
| shuifen | CV9 | Chronic diarrhea/Edema/Ascites and abdominal fullness | 1 |
| huaroumen | ST24 | Gastritis / constipation / Irritable Bowel Syndrome (IBS) / Obesity/abdominal distension/pain/digestive disorders | 1 |
| daimai | GB26 | Irregular menstruation, pelvic inflammation / Urological issues: Frequent urination, urinary tract infections/Musculoskeletal problems: Lower back pain, sciatica/Digestive complaints: Abdominal bloating, constipation | 1 |
| yangbai | GB14 | Eye disorders: Conjunctivitis, glaucoma, optic nerve atrophy/Neurological issues: Bell’s palsy, trigeminal neuralgia/Headaches: Tension-type headaches, migraines/Psycho-emotional conditions: Stress, restlessness | 1 |
| guilai | ST29 | Gynecological disorders: Endometriosis, ovarian cysts, postpartum recovery/Urological issues: Chronic cystitis, urinary retention/Digestive complaints: Lower abdominal bloating, hernias | 1 |
| laogong | PC8 | Psycho-emotional disorders: Stress, irritability, panic attacks/Oral issues: Canker sores, dry mouth/Hand disorders: Arthritis, nerve compression/Emergency use: Shock, fainting, excessive sweating | 1 |
| ezhongdai | Middle line of forehea MS1 | Anxiety, insomnia, mood disorders/Headache, dizziness, nasal congestion | 1 |
| edingdaizhong | Middle of the superior line of forehea 1/3 | Motor Regulation/Indications for stroke hemiplegia, Parkinson's disease, muscle stiffness and other movement disorders / Balance Improvement/For cerebellar ataxia, unsteady gait / Mental Relaxation/Adjunctive treatment Motor aphasia, anxiety with somatic symptoms. | 1 |
| edingdaihou | Inferior of the superior line of forehea 1/3 | Lower Limb Motor Control/Indications Lower limb paralysis, paraplegia, sciatica after stroke/Bladder Regulation/Improvement of neurogenic urinary retention/incontinence (especially sequelae of cerebrovascular disease)./Lumbosacral Pain Relief/Adjuvant treatment of lumbar disc herniation, piriformis syndrome | 1 |
| lvdidaiqian | Superior line of basis cranii 1/3 | Regulates autonomic function/It is mainly used for the treatment of abnormal blood pressure, arrhythmia, gastrointestinal disorders/Improves cranial circulation/It is used for vertebrobasilar insufficiency, tinnitus and vertigo/Relieves head and face symptoms/Treatment of trigeminal neuralgia, temporomandibular joint disorders | 1 |
| lvdidaizhong | Middle line of basis cranii 1/3 | Central-peripheral neuromodulation/It is mainly used for cervicogenic headache and occipital neuralgia/Vertebral artery supply regulation/Improve cervical vertigo and posterior circulation ischemia/Neck and shoulder syndrome treatment/Relieves upper limb numbness caused by cervical spondylosis | 1 |
| feishu | FeishuBL13 | Cough, asthma, bronchitis, pneumonia, tuberculosis, common cold/Skin issues: Acne, pruritus (since the lungs govern the skin and hair, this point can regulate skin function/ Back pain, night sweats, nasal congestion. | 1 |
| lieque | Lieque LU7 | Cough, asthma, sore throat /Common cold, headache/Migraine, tension headache, toothache/Nasal congestion, runny nose/Wrist pain, tendonitis/Urinary retention, enuresis | 1 |
| weizhong | Lieque BL40 | Acute/chronic lower back pain/Sciatica, knee pain, leg cramps/Febrile diseases, sunstroke/Skin rashes, eczema | 1 |
| quchi | Quchi LI11 | High fever, heatstroke /eczema, urticaria, acne/Elbow pain, tennis elbow, arm numbness/Hypertension/Autoimmune disorders (e.g., rheumatoid arthritis)/Constipation, abdominal pain | 1 |
| dadun | Dadun LR1 | Emotional & Neurological Disorders: 1. Stress, irritability, insomnia, Epilepsy, convulsions / 2. Urogenital Conditions Hernia, testicular pain, menstrual cramps/Urinary retention, frequent urination /3. Hemorrhage & pain uterine bleeding, nosebleeds/Toe pain, gout | 1 |
| shangxing | Shangxing GV23 | 1. Head & Facial Disorders Headaches, dizziness, Nasal congestion, nosebleeds/Red, swollen, or painful eyes / 2. Mental & Neurological Conditions Insomnia, anxiety, Epilepsy / 3. Other Uses Hair loss/Fever | 1 |
| yinjiao | Yinjiao CV7 | 1. Gynecological & Urogenital Disorders: Menstrual disorders/Leukorrhea, vaginal discharge/Urinary difficulties: frequency, retention, or incontinence / 2. Digestive Conditions Diarrhea or constipation/Abdominal distension/pain / 3. Reproductive Health Infertility/Postpartum recovery | 1 |
| jingmen | Jingmen GB25 | Low back pain/Edema, frequent urination/Abdominal distension, diarrhea/Hypochondriac pain/Chronic fatigue/Tinnitus, hearing loss | 1 |

**Supplemental** **Table S4** Subgroup analysis.

| **Subgroup** | **Change in PSQI** | | | | **Clinical efficiency** | | | |
| --- | --- | --- | --- | --- | --- | --- | --- | --- |
|  | **Study** | **SMD [95% CI]** | **p-value** | ***I*^2^** | **Study** | **RR [95% CI]** | **p-value** | ***I*^2^** |
| **Total** | 26 (28) | -1.00 [-1.21, -0.79] | <0.00001 | 82% | 22 (24) | 1.25 [1.20, 1.30] | <0.00001 | 0% |
| Intervention method |  |  |  |  |  |  |  |  |
| Only acupuncture | 12 | -0.87 [-1.15, -0.59] | <0.00001 | 76% | 8 | 1.27 [1.17, 1.38] | <0.00001 | 20% |
| Acupuncture and non-drug therapy | 3 | -1.03 [-1.57, -0.49] | 0.0002 | 70% | 4 | 1.26 [1.13, 1.40] | <0.0001 | 0% |
| Acupuncture combined with Chinese herbal medicine | 8 | -0.94 [-1.22, -0.65] | <0.00001 | 70% | 8 | 1.24 [1.16, 1.33] | <0.00001 | 0% |
| Acupuncture combined with Western medicine | 5 | -1.41 [-2.30, -0.51] | 0.002 | 94% | 3 | 1.23 [1.11, 1.36] | <0.00001 | 0% |
| Mean/median age |  |  |  |  |  |  |  |  |
| ≥50y | 14 | -1.02 [-1.30, -0.74] | <0.00001 | 80% | 9 | 1.26 [1.18, 1.35] | <0.00001 | 0% |
| <50y | 12 | -0.99 [-1.30, -0.67] | <0.00001 | 85% | 13 | 1.24 [1.18, 1.31] | <0.00001 | 0 |
| Control group |  |  |  |  |  |  |  |  |
| Traditional Chinese Medicine | 5 | -1.11 [-1.34, -0.87] | <0.00001 | 25% | 6 | 1.26 [1.15, 1.37] | <0.00001 | 0 |
| Estezolam | 11 | -0.91 [-1.29, -0.54] | <0.00001 | 89% | 11 | 1.24 [1.17, 1.31] | <0.00001 | 0 |
| Alprazolam | 1 | -0.71 [-1.23, -0.18] | 0.008 | / | 1 | 1.37 [1.01, 1.86] | 0.04 | 0 |
| Oryzanol | 2 | -1.58 [-2.17, -0.99] | <0.00001 | 68% | 3 | 1.23 [1.11, 1.36] | <0.0001 | 0 |
| Zopiclone | 1 | -0.55 [-1.19, 0.10] | 0.10 | / | / | / | / | / |
| Gua Sha | 1 | -1.00 [-1.53, -0.46] | 0.0002 | / | 1 | 1.28 [1.01, 1.61] | 0.04 | 0 |
| Sham acupuncture | 5 | -0.88 [-1.23, -0.53] | 0.00001 | 59% | / | / | / | / |
| Non-intervene | 1 | -2.21 [-2.84, -1.59] | <0.00001 |  | 1 | 2.16 [1.40, 3.35] | 0.0005 |  |
| Intervention time |  |  |  |  |  |  |  |  |
| < 4-weeks | 7 | -1.14 [-1.53, -0.75] | <0.00001 | 76% | 4 | 1.26 [1.14, 1.40] | <0.00001 | 0 |
| 4-weeks | 11 | -1.05 [-1.49, -0.60] | <0.00001 | 89% | 10 | 1.26 [1.18, 1.35] | <0.00001 | 0 |
| > 4-weeks | 7 | -0.80 [-1.02, -0.59] | <0.00001 | 57% | 7 | 1.22 [1.14, 1.30] | <0.00001 | 0 |
